# Genome-Wide Gene Expression in relation to Age in Large Laboratory Cohorts of *Drosophila melanogaster*

**DOI:** 10.1155/2015/835624

**Published:** 2015-05-21

**Authors:** Kimberly A. Carlson, Kylee Gardner, Anjeza Pashaj, Darby J. Carlson, Fang Yu, James D. Eudy, Chi Zhang, Lawrence G. Harshman

**Affiliations:** ^1^Biology Department, University of Nebraska at Kearney, Kearney, NE 68849, USA; ^2^Department of Biostatistics, University of Nebraska Medical Center, Omaha, NE 68198, USA; ^3^Department of Genetics, Cell Biology, and Anatomy, College of Medicine, University of Nebraska Medical Center, Omaha, NE 68198, USA; ^4^School of Biological Sciences, University of Nebraska-Lincoln, Lincoln, NE 68588, USA

## Abstract

Aging is a complex process characterized by a steady decline in an organism's ability to perform life-sustaining tasks. In the present study, two cages of approximately 12,000 mated *Drosophila melanogaster* females were used as a source of RNA from individuals sampled frequently as a function of age. A linear model for microarray data method was used for the microarray analysis to adjust for the box effect; it identified 1,581 candidate aging genes. Cluster analyses using a self-organizing map algorithm on the 1,581 significant genes identified gene expression patterns across different ages. Genes involved in immune system function and regulation, chorion assembly and function, and metabolism were all significantly differentially expressed as a function of age. The temporal pattern of data indicated that gene expression related to aging is affected relatively early in life span. In addition, the temporal variance in gene expression in immune function genes was compared to a random set of genes. There was an increase in the variance of gene expression within each cohort, which was not observed in the set of random genes. This observation is compatible with the hypothesis that *D. melanogaster* immune function genes lose control of gene expression as flies age.

## 1. Introduction

Aging is a general characteristic of life occurring across a great range of life forms [1]. Aging is significantly affected by genes [2]. Distantly related species exhibit similarity in the pattern of gene expression as a function of aging [3]. As may be predicted from phylogenetic and genetic conservation of aging, the mechanisms of aging may be classified into general categories. Nine hallmarks of aging are indicated in a review of the literature [4]: genomic instability, telomere attrition, epigenetic alterations, loss of proteostasis, deregulated nutrient sensing, mitochondrial dysfunction, cellular senescence, stem cell exhaustion, and altered intercellular communication. These hallmarks of aging can provide a general framework for interpreting patterns of gene expression as organisms age.

Extrinsic factors, external threats to survival, can play a major role in senescence [5]. One such factor is disease and it is known that the immune system deteriorates as a function of age in organism as diverse as humans [6] and the worm* Caenorhabditis elegans* [7]. Clearly, there is a connection between genes that affect aging and infectivity. A relationship exists in* Drosophila melanogaster* in which insulin signaling/IGF-like mutations increase lifespan and increase resistance to infection [8]. Mutations in this pathway that act to extend life span can also oppose deterioration of an aging immune system and provide resistance to infection. There are various lines of evidence indicating the importance of the immune system as an underlying factor affecting aging.

Previous research has been conducted on genome-wide gene expression in the context of aging using model systems for genetic research, especially the worm* C. elegans* and the fly* D. melanogaster*. Partially due to the degree of genetic control of the trait, the importance of investigating gene expression during the aging process has been emphasized [9]. As described above,* C. elegans* has been used for such research, for example, McCarroll et al. [3]. Moreover, relevant studies have been conducted using* D. melanogaster* specific body parts [10], quantitative trait loci [11], specific tissues [12], overexpression of a heat shock protein [13], different developmental stages [14], selection for mated longevity [15], low dose radiation [16], selection for starvation resistance [17], response to heat stress, oxidative stress, and ionizing radiation [18], lines selected for late-age fertility and life span [19], selection under hypergravity [20], and selection for postponed senescence [21]. The present study extends this category of aging research on* D. melanogaster* by relatively frequent sampling during the adult fly aging process.

Our motivation for conducting the present research using* D. melanogaster* was multifold. There is a relatively high degree of disease gene orthology between* Drosophila *and humans [22]. Thus, research on* D. melanogaster* can have human health implications. The genome of* Drosophila* is sequenced and the function of many genes studied to good degree. The life span of* Drosophila* is short and thus it is possible to study the entire aging process in a relatively short period of time. Culturing flies is well-established and it is possible to rear large numbers in a controlled environment, which was important for our research.

The present study was based on simultaneous establishment of two large populations of* D. melanogaster* used to obtain a relatively large number of adult age samples for investigation of genome-wide patterns of genes expression based on an experimental design of frequent sampling from experimental cohorts. Nine clusters of age-associated differential gene expression were identified in the present study as were a range of genes that are candidates for playing a significant role in aging. Characterization of the variance of gene expression of immune function genes as a function of age was also a focus of the present study.

## 2. Methods and Materials

### 2.1. *Drosophila* Population, Rearing, and Replicate Population Establishment

The* D. melanogaster *population used as a source of flies for this study was an outbred population kept at large numbers (approximately 15,000) for approximately 16 months in the laboratory using an overlapping generation regime (i.e., only 20% of the food bottles were replaced each week). This population was initiated from 20 lines that were inbred for 20 generations by sib-mating each generation starting from the progeny of a singly inseminated female used to start each line. As the source for this population, naturally inseminated females were collected from a natural population in the Wolfskill Orchards in Winters California maintained by the University of California at Davis. All flies were maintained on standard food consisting of cornmeal, molasses, and* Torula* yeast medium. The source population used for the present study was initiated from the 20 inbred lines using approximately 100 progenies from each of all possible crosses between the lines. This population was housed at the University of Nebraska-Lincoln. To initiate the population samples for the present study, flies in the source population were allowed to lay eggs in “pint bottles” for 48 hours at 25°C with diurnal light. After 48 hours of egg laying, 50 vials were seeded with 75 eggs each and sent overnight to the University of Nebraska at Kearney. Upon receipt, the seeded vials were placed at 25°C with diurnal light until eclosion. After eclosion, the flies were lightly etherized and separated by sex and mating sets of 25 of each sex were placed in 8 oz bottles containing food. A total of 85 bottles were prepared, and the flies in each bottle were allowed to mate for 48 hours. After the initial mating, the parent flies were transferred to a new set of 85 bottles, allowed to mate for 48 hours, and then transferred again to another set of 85 bottles for third mating. In total, there were three sets of bottles for all mating sets of 85 bottles each. The mating sets were held in a laboratory at ~22–24°C with a diurnal light cycle.

The flies were watched closely once pupation was evident and at the time of eclosion, they were lightly etherized, separated by sex, and counted. At each collection, 75 females or 75 males were placed into individual 8 oz bottles with food, until approximately 25,000 flies were collected (12,500 males and 12,500 females). The females were allowed to mature for 3 days and the males were allowed to mature for a minimum of 2 days. After this time period, sets of 75 females and males were allowed to mate for 24 hours. After mating, the flies were gently etherized, separated by sex, and counted and males were discarded. Approximately 12,000 mated females were released into a 3′ × 2′ × 1′ Plexiglas cage. Two cages were initialized in this manner. Each cage had two holes on either side covered with Tubigrip (ConvaTec, Princeton, NJ) to allow access into the cage without the loss of flies. The cages each contained six large Petri dishes of media and an additional two large Petri dishes of cotton balls moistened with Nanopure water. The cages were held in a laboratory at ~22–24°C with a diurnal light cycle. The media Petri dishes were changed every day, the water was checked every day, and water was replaced every other day. The cages had their positions changed every day with respect to top or bottom cages as they were stacked on top of each other.

### 2.2. Sample Collection and Mortality Tabulation

Each day, the dead flies were collected by aspiration and tallied. Mortality curves comparing the number of total dead flies over time were constructed. Control time point sexually mature female flies were collected at 6 days old (4 days old before being released in the boxes and after two days residency in the large cages that were sampled for the present study). Flies from this time point were used for the standard sample in the two-sample microarrays used in the present study. Over the course of this study, twenty-two samples of 24 females each were collected by aspiration, gently etherized, counted, and allowed to recover for two hours in vials containing fly food. After two hours, the females were flash-frozen in liquid nitrogen, transferred to dry ice, and stored at −80°C. Every seven days after the collection of the control females, four samples of 12 females each were collected following the same protocol as the control females. The flies were collected at 1:00 pm CST and frozen at 3:00 pm CST. Collection lasted until day 79 after introduction of the flies into the each cage. At this time point, there were only enough surviving females for this last collection.

### 2.3. RNA Extraction and cDNA Microarray Analysis

Total RNA was extracted from all sets of female flies collected at days 2, 9, 16, 23, 30, 37, 44, 51, 58, 65, 72, and 79 in the cages utilizing the standard TRIzol protocol following manufacturer's instructions (Invitrogen, Carlsbad, CA). The RNA was cleaned using the Qiagen RNeasy Mini Kit per manufacturer's instructions (Qiagen, Valencia, CA). The quality and integrity of the RNA were assessed at the UNL Genomics Core Facility by using an Agilent 2100 Bioanalyzer (Agilent Technologies, Inc., Palo Alto, CA). The RNA was quantified and cDNA microarray analyses were performed by the University of Nebraska Medical Center (UNMC) Microarray Core Facility. Two-color Version 2 DGRC oligonucleotide microarrays (*Drosophila* Genomics Resource Center [DGRC], Bloomington, IN) were used to compare gene expression over time. The microarrays consist of 15,158 oligonucleotides corresponding to roughly 93% of the annotated genes of the* D. melanogaster*. The use of two-color arrays in this experiment follows the use of the same technique in one of the very first hallmark microarray experiments to demonstrate genome-wide gene expression in* Saccharomyces cerevisiae *[23]. In addition, this technique has been employed to evaluate gene expression changes of schizont and trophozoite stages of* Plasmodium falciparum* [24].

Indirect labeling with Cy3/Cy5 fluorescent dyes was performed using 12 *μ*g of total RNA per sample using the Superscript Indirect cDNA Labeling System for DNA Microarrays (Invitrogen). All reagents and buffers used were included in the kit. Briefly, following reverse-transcription (RT), the resultant amino-allyl labeled cDNA was incubated with Cy3/Cy5 in DMSO to couple the Cy3 or Cy5 dyes to the cDNA to create fluorescently labeled probes. The CY3 and CY5 labeled probes were purified by gel-exclusion chromatography using SNAP columns (Invitrogen). Prior to hybridization, the microarray slides were prehybridized for 45 minutes at 42°C in 3X SSC solution (3 M NaCl, 0.3 M sodium citrate, 1 mM EDTA) containing 1% bovine serum albumin. The Cy3 and Cy5 probes were mixed together in 40 *μ*L Ambion hybridization buffer #2 (Ambion). Blocking agents that included poly-dA (20 *μ*g) and* Cot-1 *DNA (20 *μ*g) were added. Hybridization was performed overnight at 42°C. After hybridization, the slides were washed 2x times with 2.0x SSC, 0.5% SDS at 42 degrees for 15 minutes, followed by washing 2x with 0.5x SSC, 0.50% SDS for 15 minutes each.

Cy3 (532 nm) and Cy5 (635 nm) scans were performed using a ScanPix 4000B slide reader as per manufacturer's suggested conditions (Molecular Devices, Sunnyvale, CA). Care was given during the scanning procedure to carefully adjust the photomultiplier tubes (PMTs) such that the overall intensity from both the Cy3 and Cy5 channels was equalized. Following image capture, the overall images, as well as the individual spots, were assessed for uniformity of hybridization and individual integrity. Problematic spots (i.e., problematical morphology or those with aberrant hybridization properties) were flagged for subsequent removal from the final data set. The intensity assessment for gene spots from 16 bit TIFF files was performed with the GenePix image analysis software (Molecular Devices).

### 2.4. cDNA Microarray and Gene Ontology Analyses

The initial cDNA microarray analysis was to determine pairwise comparisons of each time point to the control (2 days in the cage, at which point the females were 6 days post-eclosion). The sample of females collected at 2 days in the cage was used as a common reference. The later-age samples from the cages were taken at 11 time points: 9, 16, 23, 30, 37, 44, 51, 58, 65, 72, and 79 days in the cages. Analyses were conducted with Linear Models for Microarray Analysis (LIMMA) package in Bioconductor [25, 26]. LIMMA uses the linear model to analyze complex experiments involving multiple experimental conditions, with an empirical Bayes approach to effectively borrow information across genes making the analyses stable even for experiments with small number of arrays. First LIMMA (a Bayesian version of linear model) from R was applied to compare the log ratio of gene expression between each of the time points and the control (day 2) after adjusting for the box effect. The Benjamini Hochberg approach was used to control the False Discovery Rate (FDR) to be less than 0.05 [27]. A gene was deemed to be differentially expressed if and only if under at least one time point the Benjamini Hochberg adjusted *p* value was no more than 0.05 and the raw fold change was ≥1.5 or ≤1/1.5. The box effects were partially removed by normalizing the relative intensities with a zero median. In addition, the box effects were adjusted on each gene when evaluating the differential gene expression on that gene. All of the data has been submitted to NCBI: Gene Expression Omnibus at http://www.ncbi.nlm.nih.gov/geo/ (GSE67547).

The genes identified as differentially expressed across all the time points were subjected to clustering analyses. Clustering is a powerful exploratory technique for the analysis of gene expression data. The underlying biological assumption for clustering of genes is that genes participating in the same biological process are expected to exhibit similar expression patterns. A self-organizing map (SOM) clustering algorithm [28] was applied to the significantly differentially expressed genes. These SOMs are somewhat related to *k*-means clustering but allow users to impose partial structure on the clusters. Specifically, the users need to prespecify the geometry of nodes (i.e., a 2*∗*2 grid), and the nodes will be iteratively mapped into *k*-dimensional gene expression space. The data points with close distance will be grouped into the same node and the neighboring nodes of the SOMs tend to define related clusters. Tamayo et al. (1999) stated that the SOMs provide easy implementation, visualization, and interpretation with superior robustness and accuracy [28]. We considered the available options for the geometry of nodes and select the 3*∗*3 grid that the other settings with more nodes will not produce fundamentally new patterns, and reasonable genes were classified into each SOM cluster. In addition, the average fold changes at each time point (when compared to day 0) of all genes within each cluster have different patterns that have different signs of the fold changes and moderate (fold change > 1.2) or large fold change (fold change > 1.5) at different time points. The mean log_2_ ratios between any other time point and time point 2 days for these identified genes were estimated and used as gene expression profiles in the clustering analysis. The profile for each gene was standardized so that each profile had a mean = 0 and a standard deviation = 1. Data matrices were constructed with genes in rows and time points in columns. The GeneCluster 2 package was used [28]. The number of iterations in SOM clustering was set to 500,000. From this, self-organizing map (SOM) and hierarchical clustering heat map (correlation-based distance, average link) were generated. This type of analysis allowed each gene to be uniquely clustered into one of the nine clusters. The identified differentially expressed genes were selected for further analyses. These genes were subjected to ontology analyses using PANTHER (Protein Analysis through Evolutionary Relationships; http://www.pantherdb.org/) [29–31].

### 2.5. Gene Set Enrichment Analysis (GSEA)

Light intensity observations from the scanned image of five replicates were subjected to quality assurance as implemented in various Bioconductor packages [32]. Background correction, normalization, empirical Bayes correction, and the calculation of statistical significance for differential gene expression were performed by using the LIMMA package [25]. For multiple test correction, Benjamini and Hochberg's False Discovery Rate was used [27].

High-level overviews of the biological processes affected by the transcriptional dynamics of aging were obtained by using comprehensive classifications systems. These systems include the KEGG (Kyoto Encyclopedia of Genes and Genomes) Database of Biochemical Pathways [33] and the Gene Ontology (GO) categories for biological processes, molecular functions, and cellular localizations, originally developed by* Drosophila *experts [34]. Gene sets, such as pathways, GO categories, or genes regulated by a particular transcription factor, allow examination of transcriptional changes at levels much higher than the level of single genes. While, for example, none of the individual genes of a pathway are induced at some level of statistical significance, a consistent but possibly marginal upregulation in the majority of the genes may be biologically more relevant than a major induction of just a few genes. The statistical significance of enrichment of a gene set in either the upregulated or downregulated genes is calculated by using Gene Set Enrichment Analysis (GSEA) [35]. GSEA applies a nonparametric permutation test that does not rely on the normality of the fold change value distribution. It is a high-performance analysis method that can accurately integrate transcriptomics or proteomics results into the context of gene ontology or biochemical pathways. To this end, in-house PERL libraries to transform LIMMA output and annotations to GSEA input as well as for postprocessing of GSEA outputs were used.

Transcript level patterns across the seventy-nine day time span of the experiments were assessed by* k*-means cluster analysis using different numbers of clusters. The consistency of transcript level changes through time was evaluated by the MATLAB implementation of the biclustering method, that is, clustering the base 2 logarithm fold change values both by genes and transcript levels [36]. Biclustering, with minor exceptions, faithfully reproduced time points in chronological order.

### 2.6. Gene Expression Variance

Variation in gene expression was calculated for two sets of genes. Immune function genes were a focus for the measurement of variance as they were relatively frequently represented among the genes that exhibited differential expression as a function of age in the present study. Immune function gene samples were selected from a website in a publication describing such genes for* D. melanogaster* [37]. Based on the correspondence between the genes in this website and the probes in the microarrays used in the present study, 316 genes were included in the analysis. The measure of variance was based on the three samples that were taken for each time point used for the present study. Variance in expression of immune genes was calculated within cages (boxes A and B), and variance among cages, which was based on a mixture of samples from box A and box B. Measurement of the variance of gene expression within cages allowed for insight into the pattern of gene expression that was responsible for identification of this class of genes identified by earlier analysis of the data including PANTHER and GSEA clustering. Statistical mixing of the samples from different cages was conducted; these samples were used to calculate the slope of change in gene expression which allows for an assessment of a potential cage (box) effect as an environmental variable. For comparison to immune function genes, a set of 200 genes was randomly selected using the entire list of* D. melanogaster* genes, minus immune function genes. This set of genes was analyzed as representative of the genome of* Drosophila*. A random number generator was used to selected genes from this list. Variation was calculated as the standard deviation of average gene expression. A linear model was fitted between the variance of expression and time points using SAS [38].

### 2.7. Assessment of cDNA Microarrays by qRT-PCR

Reverse transcription was performed using Taqman Gene Expression Assay kits and the 7500 Real Time PCR system (Applied Biosystems, Foster City, CA) according to manufacturer's instructions. The primer and probe sets used were* Metchnikowin *(*Mtk*; assay #Dm01821460_s1),* CG11671* (assay #Dm02137913_g1),* Neuropeptide-like precursor 3 *(*Nplp3*; Dm02369807_s1),* Retinin *(*Rtn*; Dm02363430_s1), and* Ribosomal protein 49 *(*Rp49; *endogenous control; assay Dm02151827_g1). Reactions were carried out in triplicate and performed in a 50 *μ*L volume utilizing 200 ng total RNA sample and TaqMan One-Step RT-PCR Mix (Applied Biosystems). Negative controls without RNA for each primer/probe set were also run. Cycling parameters included 48°C for 30 minutes, 95°C for 10 minutes, and 40 cycles of 95°C for 15 seconds and 60°C for 1 minute. The PCR products were analyzed in the linear range for amplification with* Rp49* using the 7500 Real Time PCR System Sequence Detection Software (Applied Biosystems). The relative quantitative results were used to determine changes in gene expression on a log_2_ scale.

## 3. Results

### 3.1. SOM/Clustering and PANTHER Analysis

**Figure 1:**
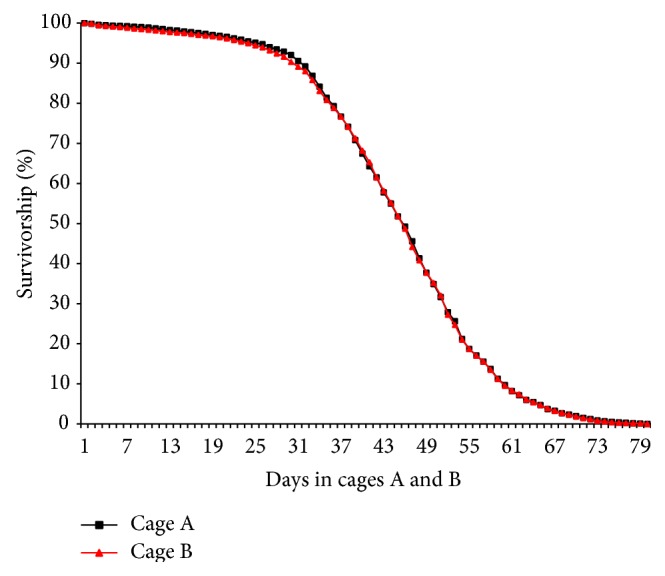
Percent survivorship females from cages A and B over the course of the experiment (day 79 in the cages). Peak of death occurs near day 31. The *x*-axis is the number of days the* D. melanogaster* remained inside the boxes. The *y*-axis shows the percent survivorship of the population at each day of the experiment. The cages were each initiated with ~12,000 once mated females. An overall decrease in survivorship was observed between days 30 and 60.

Each day, dead flies were collected by aspiration, separated by sex, and tallied. Mortality curves comparing the number of total dead flies over time are shown in Figure 1. The two cages demonstrate almost completely overlapping curves, with death starting at approximately day 30. Between days 30 and 60, there is a noticeable decrease in overall survivorship in both cages (Figure 1). Microarrays were based on a comparison of days 9, 16, 23, 30, 37, 44, 51, 58, 65, 72, and 79 in the cages to the control time point (two days in the cages). After filters and normalization were applied, 1,581 significantly changed genes were found in comparisons to the day 2 control. The cDNA microarrays were validated by qRT-PCR using two upregulated genes,* Metchnikowin* (*Mtk*) and* CG11671*, and two downregulated genes,* Retinin* and* Neuropeptide-like precursor 3 *(*Nplp3*). The data from these genes was compared with the cDNA microarray data for fold change at days 16 and 79. The patterns of gene expression (up- or downregulated) showed good correspondence between microarrays and qRT-PCR (Table 1).

**Figure 2:**
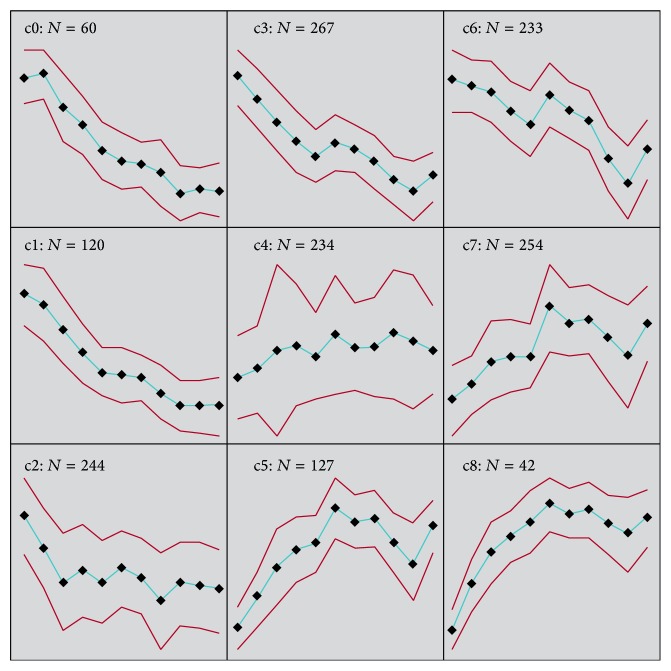
Three rows by three columns self-organizing map (3 × 3 SOM) for gene expression patterns of clusters. Each cluster is represented by the centroid (average pattern) for genes. The *x*-axis shows the time points (days in the cages). The *y*-axis is the standardized mean log_2_ ratios between any other time point and time point 0 and indicates average fold change. The number of genes within each cluster is represented by the whole number in the top center of the square. For example, in the top left-hand corner is cluster 0 and the number 60, which indicates *n* = 60 genes. Overall, cluster 0 (*n* = 60), cluster 1 (*n* = 120), cluster 2 (*n* = 244), cluster 3 (*n* = 267), cluster 4 (*n* = 234), cluster 5 (*n* = 127), cluster 6 (*n* = 233), cluster 7 (*n* = 254), and cluster 8 (*n* = 42). The first black diamond represents day 9, followed by days 16, 23, 30, 37, 44, 51, 58, 65, 72, and 79 as the remainder of the sampled days. The red lines on either side of the blue line connecting the black diamonds represent the confidence interval.

The 1,581 genes differentially regulated at one or more time points (see Supplementary Table 1 in Supplementary Material available online at http://dx.doi.org/10.1155/2015/835624) were submitted to PANTHER analysis and 525 of the IDs were unrecognized. The PANTHER Classification System is a resource that classifies genes by their functions, using published experimental evidence and evolutionary relationships to predict function even in the absence of direct experimental evidence [28, 29]. Of the remaining 1,061 genes, the selected annotated biological functions (gene ontology accession number; percent of gene hit against total number genes; percent of gene hits against total number of process hits) included various roles in cell communication (GO: 0007154; 19.5; 9.8), cellular processes (GO: 0009987; 26.4; 13.3), localization (GO: 0051179; 0.5; 0.2), transport (GO: 0006810; 16.3; 8.2), cellular component organization (GO: 0016043; 5.9; 3), apoptosis (GO: 0006915; 4.0; 2), system processes (GO: 0003008; 15.1; 7.6), reproduction (GO: 0000003; 4.3; 2.1), response to stimulus (GO: 0050896; 8.2; 4.1), homeostatic processes (GO: 0042592; 0.7; 0.3), developmental processes (GO: 0032502; 12.1; 6.1), generation of precursor metabolites and energy (GO: 0006091; 4.0; 2), metabolic processes (GO: 0008152; 56.3; 28.4), cell cycle (GO: 0007049; 6.4; 3.2), immune system processes (GO: 0002376; 12.8; 6.4), and cell adhesion (GO: 0007155; 6.2; 3.1). Clustering analysis was applied to the 1,581 significant genes to determine their expression patterns over time. From these analyses, a 3 × 3 SOM (Figure 2) was created. The genes present in each cluster represent a gene that is significantly changed at any one time point during the time course compared with the day 2 time point control, but not necessarily differentially regulated at every time point. Each cluster of genes uncovered in the 3 × 3 SOM analysis was analyzed via PANTHER to determine gene ontology groups and selected biological functions (Table 2). In all the clusters, the category that is most largely represented is metabolic processes (GO: 0008152). The gene ontology category that is least represented is localization (GO: 0051179), with homeostatic processes (GO: 0042592) also being underrepresented across all SOM clusters at all time points (Table 2).

The number of genes changing per time point for each cluster was determined and found to fluctuate to varying degrees, with the exception of SOM clusters 0 and 8 (Table 3) that remained fairly consistent in the number of genes being expressed. The only cluster to show a change in gene expression at day 9 was cluster 0, which had* CG9297 *and* Retinin* downregulated −1.59- and −2.16-fold, respectively, compared to the day 2 control. The consistency of cluster 0 begins at day 37 with 95% of the genes in this cluster being downregulated and 98.3–100% downregulation achieved and maintained by day 44. For cluster 8, the consistency begins at day 23 with 88.1% of the genes being upregulated and 95.2% upregulation of all the genes in the cluster achieved and maintained by day 30 (Table 3). The distribution of genes at the latest time point, day 79, was analyzed (Table 4). The genes were separated into groups of those upregulated, downregulated, or not affected at day 79. SOM classes 0 and 8 demonstrated 100% downregulation or upregulation, respectively, at this time point compared to the day 2 control (Table 4).

Clusters 0 and 8 are relatively consistent in number of genes differentially expressed across all time points (Tables 3 and 4). Cluster 0 demonstrated a downward trend in gene expression levels with 60 genes being differentially expressed. When applied to PANTHER analysis, 31 genes from this cluster were not found. In cluster 0, there is a large representation of genes involved in metabolic processes (45%), as compared to other biological functions (Table 2), with 61.1% of these being part of protein metabolic processes (GO: 0019538). A majority of the genes in this group have functionality as serine proteases (CG32523, CG18180, and CG5246) or are part of a family of serine proteases (*Jonah 44E *[*Jon44E*],* Jon99Ciii*,* Jon25Bii*,* Jon25Biii*, and* Jon99Fi*). Of the 31 unmapped gene IDs in cluster 0, 16 have unknown biological function, 11 are involved in eggshell chorion assembly (*Chorion Proteins *[*Cp*]* 7Fa*,* 7Fc*,* 16*,* 19*,* 36*, and* 38*,* and Vitelline membrane *[*Vm*]* 26Aa *and* 34Ca*), 2 are involved in cuticle formation (*Tweedle *[*Twdl*]* M* and* F*), 1 is involved in neuropeptide signaling pathways (*Nplp3*), and 1 is involved in viral reproduction (*CG32642*). In contrast, cluster 8 exhibits upregulation in the 42 genes found in this group, specifically starting at day 30 (Tables 3 and 4). When applied to PATHER analysis, 27 of the gene IDs were not found or mapped. As with cluster 0, the metabolic processes GO group is highly represented (25.6%). Upon further analysis of the 27 unmapped IDs, 16 have unknown biological function, 9 are involved in immune system processes, specifically immune response (*Attacin *[*Att*]*-B*,* C*, and* D*,* Cecropin *[*Cec*]* A2 *and* C, Diptericin *[*Dpt*] and* DptB, drosomycin-2 *[*dro2*], and* Mtk*), and 2 are involved in stress response (*Turandot *[*Tot*]* A *and* Turandot C*). PANTHER analysis failed to assign a GO group for 9 genes involved in this GO group. When the percent of gene hits against total number of process hits for the immune system processes GO is considered; there are 11 of the 42 cluster 8 genes (26.2%) in this group, making the immune system the most represented GO process group for cluster 8.

Similar to cluster 0, clusters 1, 2, 3, and 6, also demonstrated a relative trend of downregulation of gene expression over time (Figure 2). Cluster 1 contained 120 genes and when submitted to PANTHER, 78 gene IDs were unmapped. Of the remaining 42 genes that were mapped, once again a large group of proteases (*Trypsin epsilon *[*εTry*], *θTry*,* CG1304*,* CG3088*,* CG6508*,* CG7542*,* CG8560*,* CG8562*,* CG17633*,* CG31926*,* CG31928*, and* CG32483*) and more members of the Jonah serine protease family (*Jon65Ai*,* Jon65Aii*,* Jon99Ci*,* Jon99Fii*, and* Jon74E*) were found. Members of the protease group were found in clusters 2 (*Angiotensin-converting enzyme-related*,* CG1503*,* CG3775*,* CG5794*,* CG5839*,* CG6726*,* CG7025*, and* CG8539*), 3 (*CG3734*,* CG4847*,* CG4914*,* CG5240*,* CG6733*,* CG8299*,* CG8774*,* CG9675*,* CG10587*,* CG11911*,* CG16749*,* CG17475*,* CG18179*,* CG31269*,* CG31661*,* Jon65Aiii*,* Jon65Aiv*,* Neprilysin 2*,* NnaD*,* nudel*,* scarface*,* Ser7*, *αTry*, *ιTry*, *ζTry*, and* yippee interacting protein 7*), and 6 (*Ance-4*,* CG3344*,* CG3739*,* CG6592*,* CG8586*,* CG9897*,* CG10081*,* CG10472*,* CG10477*,* CG10469*,* CG17012*,* CG17571*,* CG18493*,* CG18585*,* CG32834*,* prolyl-4-hydroxylase-alpha EFB*, and* Ser6*). Among these three clusters, cluster 2 contained 244 genes with 59 unmapped IDs, cluster 3 contained 267 genes with 65 unmapped IDs, and cluster 6 contained 233 genes with 63 unmapped IDs.

One cluster, cluster 7, exhibited a similar pattern of gene expression to cluster 8 (Figure 2). This cluster contained 254 genes with 104 unmapped IDs when submitted to PANTHER. Of the 254 mapped IDs, 84 genes were involved in metabolic processes and 28 others were involved in immune system responses (*Alk*,* Antigen 5-related*,* Atf6*,* CG3640*,* CG6429*,* CG6495*,* CG10089*,* CG10345*,* CG16713*,* CG16799*,* CG18480*,* CG30273*,* GstD2*,* GstD9*,* Heat shock protein 23*,* lectin-24Db*,* Lipase 4*,* Neuropeptides capa receptor*,* p38c*,* Peptidoglycan-recognition protein-LB* [*PGRP-LB*],* PGRP-LF*,* Protein shifted*,* Tetraspanin 42Eb* [*Tsp42Eb*],* Tsp42Ed*,* Tsp42Ei*,* Tsp42Er*,* Thiolester containing protein II*, and* tolkin*).

The only cluster that did not change in an obvious up- or downexpression pattern over time was cluster 4 (Figure 2). This cluster contained 234 genes with 85 unmapped IDs when submitted to PANTHER. As with the other clusters, the largest GO category represented was metabolic processes (21%; Table 2). From the 85 unmapped IDs, 61 are of unknown function. The remaining 24 genes are variable functions including behavioral processes (*RhoGAP18B*), cellular processes (*CG3223*,* CG5466*,* Cytochrome c distal*, and* milton*), central nervous system development (*jing*), chitin metabolic processes (*CG13075* and* Mucin 11A*), dosage compensation (*Suppressor of variegation 3–7*), immune response (*Bre1* and* CG10433*), mesoderm development (*Myosin 31DF*), neuron projection morphogenesis (*Down syndrome cell adhesion molecule 2*), potassium ion transport (*CG42732*), protein folding (*CG31287*), proteolysis (*CG15157 *and* CG42264*), reproductive processes (*Accessory gland-specific peptide 26Ab*,* bag of marbles*,* CG17097*,* closca*,* hopscotch*, and* stonewall*), and response to DNA damage (*Breast cancer 2, early onset homolog*).

### 3.2. Temporal Gene Set Enrichment Analysis (GSEA)

Gene Set Enrichment Analysis (GSEA) was used to classify overrepresented categories of genes for each time point (days 9, 16, 23, 30, 37, 44, 51, 58, 65, 72, and 79) relative to the control time point (day 2 in box), characterized by gene ontology. The entire set of genes that were differentially expressed in at least one time point was used for this analysis. Overrepresented gene categories were similar to the analysis previously done. An upregulation of the immune system GO categories was found from days 16 to 79 (Table 5), as was seen in SOM clusters 7 and 8 from analysis of the unmapped IDs from the PANTHER analysis. These categories were consistently upregulated from day 16 or 23 to day 79 in the box. The most consistent downregulation was seen in phototransduction genes (GO: 0007602). Two groups of genes that are involved in eggshell formation or assembly (vitelline membrane formation involved in chorion-containing eggshell formation GO: 0007305 and eggshell chorion assembly GO: 0007306) were consistently downregulated from day 30 to day 79 in the box, which is consistent with the PANTHER analysis of the SOM clusters.

The Kyoto Encyclopedia of Genes and Genomes (KEGG) pathways was also considered using GSEA (Table 6). There was only one fairly consistent pathway that changed across all time points, which was the downregulation of the fly phototransduction pathway (04745). At the three latest time points (65, 72, and 79 days in the box), the largest proportion of differentially changed pathways was observed. At day 65 in the box, there were seven downregulated pathways including glycolysis (00010), alanine and aspartate and glutamate metabolism (00250), pyruvate metabolism (00620), ribosome (03010), starch and sucrose metabolism (00500), galactose metabolism (00052), and phototransduction (04745). Both up- and downregulated pathways were detected at day 72 in the box, including DNA replication (03030), mismatch repair (03430), base excision repair (03410), glycosaminoglycan biosynthesis (00534), ECM-receptor interaction (04512), pyruvate metabolism (00330), oxidative phosphorylation (00190), ribosome (03010), starch and sucrose metabolism (00500), galactose metabolism (00052), and phototransduction (04745). At the latest time point, 79 days in the box, 5 pathways were downregulated, which include neuroactive ligand-receptor interaction (04080), arginine and proline metabolism (00330), ribosome (03010), starch and sucrose metabolism (00500), galactose metabolism (00052), and phototransduction (04745).

Shared expression patterns of differentially expressed genes were investigated using biclustering analysis of gene expression and experiments/replicates. Days 9–79 in the box were compared to day 2 (5 days old) in the box. The data demonstrates the reproducibility of the experiments. One cluster of genes is upregulated at early time points, while being downregulated at the later time points. Another cluster of genes shows the opposite trend. The data are clustered into three clusters, early, midlife, and late. The early time points include four time points (9, 16, 23, and 30) with days 9 and 16 being closely related and days 23 and 30 being closely related. The midlife time points (days 37, 44, 51, and 58) demonstrate that days 44 and 51 are closely related and days 37 and 58 are closely related. The late time points (65, 72, and 79) are closely related. The significantly up- or downregulated genes across time points compared to day 2 in the box were determined. In order to be included, the gene had to be detected at only one time point. The GSEA detected 263 upregulated genes (Supplementary Table 2) and 355 downregulated (Supplementary Table 3) genes. Of these 618 differentially regulated genes, only 1,* Retinin*, was significantly changed (downregulated) at all 11 time points tested. Most of the genes were differentially expressed at only one time point (upregulated = 92, downregulated = 108). Genes involved in immune system regulation (*AttB*,* AttC*,* AttD*,* CecA2*,* CecB*,* CecC*,* CG2056*,* Dpt*,* dro2*,* Immune induced molecule 2 *[*IM2*],* IM3*,* IM4*,* IM23*,* Mtk*,* PGRP-SB1*, and* PGRP-SD*) were found to be upregulated across multiple time points. The downregulated genes across multiple time points included the* Jonah *family of serine proteases (*Jon25Bii*,* Jon25Biii*,* Jon44E*,* Jon65Ai*,* Jon65Aii*,* Jon99Ci*,* Jon99Ciii*, and* Jon99Fi*) and genes involved in chorion formation and assembly (*CG18349*,* CG18777*,* CG18779*,* Cp7Fa*,* Cp7Fb*,* Cp15*,* Cp16*,* Cp18*,* Cp19*,* Cp36*,* Cp38*,* Dec1*,* Fcp3C*,* Lcp65Ac*,* Lcp65Ae*,* Lcp65Ag1*,* Lcp65Ag2*,* Lsp2*,* Vitelline membrane *[*Vm*]* 26Aa*,* Vm26Ab*,* Vm26Ac*,* Vm34Ca*,* yolk protein 2* [*Yp2*], and* Yp3*).

### 3.3. Variation in Gene Expression

**Figure 3:**
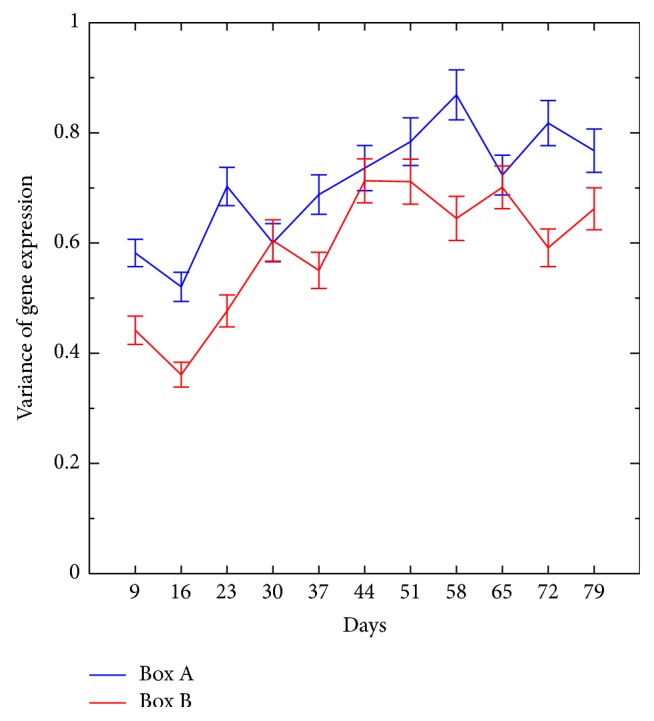
Change in the variation of gene expression of immune function genes within cages (boxes A and B). This figure presents the average variation over time. The slope of gene expression change is shown on the *y*-axis and sampling points used for transcriptome analysis on the *x*-axis.

**Figure 4:**
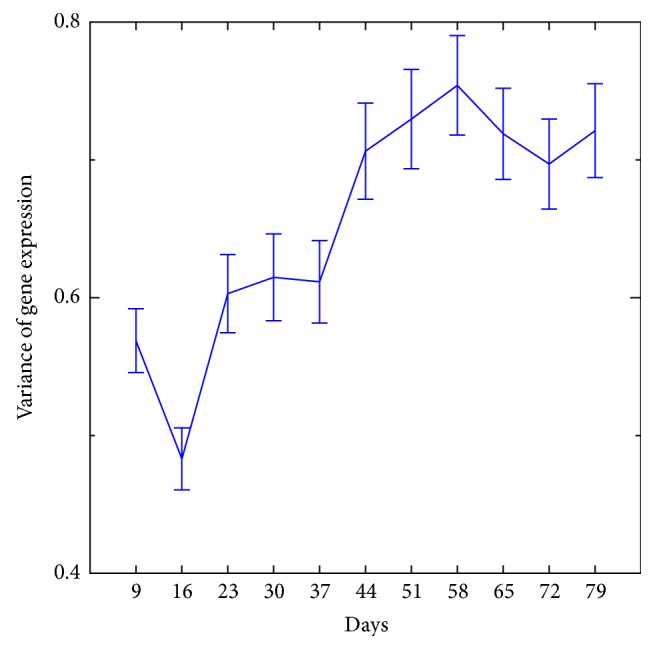
Change in the variation of gene expression of immune function genes among cages. For this analysis, samples from cages (boxes A and B) were mixed then the slope calculated. The slope of gene expression change is shown on the *y*-axis and sampling points used for transcriptome analysis on the *x*-axis.

**Figure 5:**
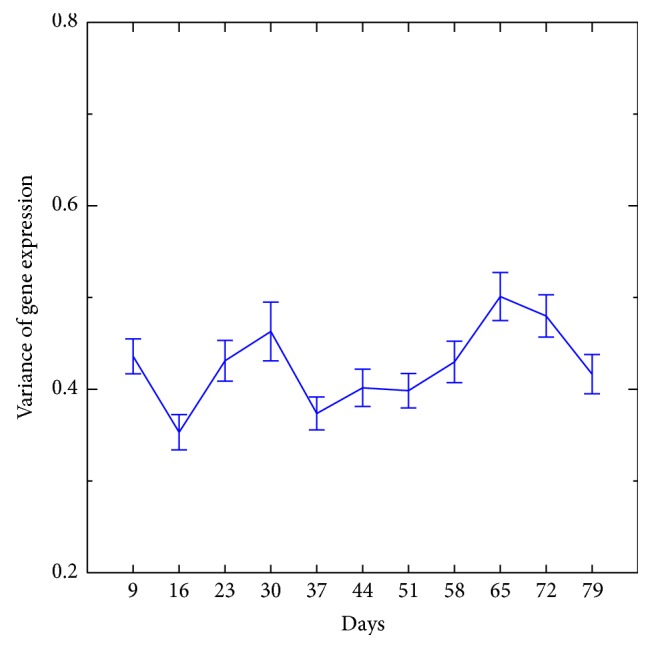
Change in gene expression of a set of 200 genes randomly selected from the* Drosophila* genome. The slope of gene expression change is shown on the *y*-axis and sampling points used for transcriptome analysis on the *x*-axis.

Figure 3 shows the effect of age on variance in immune system gene expression between samples within boxes A and B (boxes are equivalent to cages). The variation (heterogeneity) increases as a function of age. With a linear regression fitting, the slopes were 0.0417 for box A and 0.0455 for box B, and the fitting was significant: the *p* values for boxes A and B were 2.4913 × 10^−13^ and 1.89 × 10^−17^, respectively. The slope of the change in gene expression generated from samples mixed among boxes was 0.0335 and the *p* value was 1.448 × 10^−12^ (Figure 4). The slope of the gene expression among boxes did not differ from the slope within boxes.  Figure 5 shows the slope of a set of 200 randomly selected genes, which was −0.0026. The slope of this random set of genes was as not statistically significantly different from an absence of slope (*p* < 0.5318). Presumably, this randomly selected set of genes was representative of much of the genome.

## 4. Discussion

In the present study, we conducted a fine-scaled temporal analysis of genome-wide gene expression in replicate laboratory populations. The only factor that obviously varied over the course of the study was age, but the environment could have varied from one cage to the other. The data in the present study was generated from replicate populations, initially very nearly 12,000 mated female* D. melanogaster. *The mortality curves were generated by counting the number of dead females every day for the length of the study, which was until all flies were dead. The mortality curves demonstrate a decrease in survivorship starting at day 30, with a steady decline until day 60. The LT_50_ of survival for each cage was 45 days (Figure 1). When comparing the two mortality curves, the replicates essentially overlap at every time point. This result suggests a high degree of similarity in the two study populations. The high degree of similarity of slope of change in gene expression (described in Figures 3, 4, and 5) also supports the conclusion that the environment in the* Drosophila* cages was quite similar.

Our data set included 1,581 genes that were differentially expressed as a function of age as revealed by SOM clustering and PANTHER analysis. There was consistency among clusters in the pattern of gene expression. As one example, clusters 0, 1, 2, 3, and 6 exhibited a marked decline in gene expression starting the first week of life. Cluster 2 showed a leveling off of this decline starting the second week of life. Clusters 0 and 1 consisted of genes whose expression levels off late in life. Clusters 3 and 6 are apparently characterized by an upturn in level of gene expression late in life. As another example of consistency, clusters 5, 7, and 8 exhibited an increase in gene expression starting early in life. In these clusters, the level of gene expression levels off at approximately midlife and there was a trend of declining gene expression after the midlife leveling off, followed by an upturn of gene expression late in life. Another general pattern observed in the data was that there was an apparent tendency for an upturn in gene expression in five clusters (3, 6, 5, 7, and 8). Cluster 4 was unique in the tendency of genes in this group not to vary as a function of age. Although many genes may change expression as a function of age in* D. melanogaster*, there is no necessary loss of control of gene expression underlying changes in mRNA abundance over time [39].

Within the present study data set, there were a number of SOM clusters of interest, for example, clusters 0 and 8, due to their consistent number of differentially expressed genes throughout the life span (Tables 3 and 4). Cluster 0 showed a pattern of decline in gene expression over time as compared to the day 2 control time point (Figure 2). When the genes in this cluster were mapped over time, 57 of the 60 genes in this cluster were downregulated by day 37 (Table 3), which is over a week ahead of the LT_50_. At the LT_50_ (day 45), 59 of the 60 genes in this cluster are consistently downregulated, with the exception of* Arrestin 1 *(*Arr1*), which promotes light-induced rhodopsin endocytosis [40]. This gene was consistently downregulated beginning at day 23. At the day 45 time point,* Arrestin *was downregulated −2.53-fold compared to control day 2 with a *p* value of 0.0015. This finding is similar to that of genes involved in synaptic transmission that are downregulated in heads of aged flies [10]. The only other day with a discrepancy in this cluster is day 58, in which* CG13060* was not significantly downregulated (−3.06 fold change, *p* value = 0.127). This gene has unknown biological and molecular functions but was implicated as a miR-8 target site in a screen to identify genes upregulated in* miR-8* mutants. These* miR-8 *mutants showed reduced pupal and adult survival, in fact ~80% failed to eclose and the others died within 24 hours [41]. If* CG13060 *is a functional miR-8 target, then downregulation of this target site might contribute to the normal aging process or possibly extend life span. This gene should be further studied for functional roles in the aging process. In cluster 0, 18.3% of the genes are involved in eggshell chorion assembly and formation. This was consistent with the GSEA of GO groups that also showed a significant downregulation of this group over time (Table 5). During aging, some biological processes, such as egg formation, are expected to decline and were evident in this study with the genes involved in eggshell chorion formation decreasing steadily across time. This is consistent with another genome-wide transcriptome study [42] and a study on gene expression during egg development before and after reproductive diapause [43]. The age at when these genes changed expression was not provided in these studies, but in the present study, the SOM and PANTHER analysis demonstrated that they became consistently downregulated as a group starting at day 37. The exception was* Cp7Fa*, which did not achieve constancy until day 44 (Supplementary Table 1) and this was also observed in the GSEA data (Supplementary Table 3). Phototransduction was the other GO and KEGG group that showed consistent downregulation over time (Tables 5 and 6). This observation is in line with other studies that showed this group topping the list of significant GO terms [10, 15, 44, 45]. Also within cluster 0, the largest group of genes were involved in metabolic processes involving serine proteases. Serine proteases and serine protease homologs play diverse roles in multiple biological processes, including digestion, development, and the immune response [46]. Serine proteases and related homologs are important in development, immune responses, and other biological functions [47]. In humans, the correlation between proteolytic enzyme activity and age-related pathology suggests a possible association with physiopathological aging. A study with rats demonstrates the activity of three serum serine proteases as possible biomarkers of aging [48]. In the current study, a family containing serine protease genes, the* Jonah *family, was uncovered by both SOM and PANTHER analysis (Supplementary Table 1) and GSEA (Supplementary Table 3). The members of the* Jonah* family are expressed both during the larval stage and a few hours after eclosion throughout adult life.* In situ *hybridization localized the* Jonah *mRNAs in the midgut [49] and some Jonah proteins may aid in digestion [50]. Although the functions of these proteins in the midgut are largely unclear, this family of genes has been identified and is downregulated in other* Drosophila* aging transcriptome studies [51, 52]. In addition to digestive functions, the gut plays a role in immune homeostasis. Two effector mechanisms exist in the gut, including reactive oxygen species (ROS) generation and antimicrobial peptide (AMP) production. Alternative Imd (immune deficiency pathway) regulation and stimulation of the* duel oxidation* (*duox*) gene to produce ROS provides innate immunity within the gut [53]. In a study evaluating the correspondence of gene expression patterns in both aging and oxidative stress, the majority of known immune response genes were induced and a large number of proteases were decreased both in aging and in response to oxidative stress [18]. In* D. melanogaster*, only twenty-two genes are classified as GO: serine-type endopeptidase and GO: defense response, which makes the relationship between serine proteases and immunity unclear [46].

In our study, a large number of immune-related genes were overrepresented in PANTHER analyses (Table 2) and in the gene ontologies derived from GSEA of temporal gene expression (Table 5). The upregulation of immune function genes has been seen in other studies on* D. melanogaster* aging gene expression. Age-related upregulation of the immune genes and functional decline in immunity (immunosenescence) has been documented in other studies [reviewed in [54, 55]]. This age-related immune status is termed inflammaging [56]. This phenomenon is not restricted to* Drosophila*, as it was first characterized in humans with the observation that peripheral blood mononuclear cells from elderly individuals produce greater amounts of proinflammatory cytokines as compared to younger controls [57]. In fact, this phenomena has been noted in a variety of species and may be an underlying evolutionarily conserved mechanism of aging [58].

In addition to the present study, other studies have demonstrated upregulation of immune genes and downregulation of serine proteases, specifically the* Jonah* genes, and in response to sigma virus infection [59] and xenobiotic responses [60]. Neither of these studies had digestion as a variable; therefore the idea that the* Jonah *genes produce proteases that only deal with digestion should be questioned. Another study demonstrated an upregulation of transcript levels of immune-related genes and a downregulation of genes involved in the aging of the gut [12]. These studies suggest a connection between the gut and the immune system, possibly between the downregulation of serine proteases and the upregulation of immune response genes, with aging. In a computational annotation of serine proteases and immune response, 94 out of 2201 trypsin-like serine proteases were identified as potentially involved in the* Drosophila *immune response [47]. Contrary to the model, the serine proteases predicted to be upregulated with aging were found to be downregulated in the present study. One hypothesis is that as aging persists, there is an accumulation of oxidized, cross-linked, or aggregated proteins that cannot be processed by proteolytic systems. In turn, a critical level of these unprocessed proteins is reached and an immune response is induced [57]. Our study indicated that this relationship may occur at about the LT_50_ (day 45), or even earlier (Table 3). This suggests that the role of serine proteases and the* Jonah* family of genes and the relationship with the immune related genes should be further investigated as possible biomarkers of aging and as candidate genes for identification of the underlying pathophysiological mechanism of aging. The* Jonah* family of genes considered solely on the basis of their general protease activity may be important as foci for future research in relation to loss of proteostasis as a hallmark of aging [4].

Other candidate genes were identified in the present study using the gene lists generated from both SOM clustering (Supplementary Table 1) and GSEA for overrepresented genes (Supplementary Tables 2 and 3). These were compared to published literature and two databases: GenAge: The Ageing Gene Database (http://genomics.senescence.info/genes/index.html) [61] and Genesdb (http://www.uwaging.org/genesdb/). Of the overrepresented upregulated genes (Supplementary Table 2), the first is* drop dead* (*drd*), which was found in SOM cluster 1 (Supplementary Table 1). Earlier aging studies found that the* drd* mutants die early in adult life [62] and may cause an acceleration of the normal aging process [63]. In SOM cluster 2 (Supplementary Table 1),* Phosphoenolpyruvate carboxykinase* (*Pepck*) and* Insulin-like peptide 4* (*Ilp4*) were found to be candidate genes. In* Drosophila, Pepck* is major anaplerotic source of oxaloacetate, which is an intermediate of the citric acid cycle and gluconeogenesis [64]. Mice with overexpressed* Pepck-C* had an extended life span relative to control animals and evidently such mice repattern energy metabolism leading to greater longevity [65].* Insulin-like peptide 4 *(*Ilp4*) is part of a group of five insulin-like peptide proteins that are highly homologous to those found in mammals [66]. Like the* Jonah* genes, it is expressed in the midgut, but also in the neurons [67]. In* Drosophila *with the insulin-like peptide-producing median neurosecretory cells ablated and reduction of the* dIlp* genes, there was an extension of median and maximal lifespan [68]. In SOM cluster 3 (Supplementary Table 1), there were two genes of particular interest. The first is* sugarbabe, *which encodes a predicted zinc finger protein regulating neurosecretory cell insulin gene expression. In* miR-14* mutant flies,* sug *was found to control* dIlp* mRNA levels, suggesting that it may act as a transcription factor to these genes [69]. In another study, a 5–9% increase in mean life-span was achieved with* sug *overexpression from a doxycycline-inducible promoter [18]. Of the overrepresented downregulated genes (Supplementary Table 3), two genes from SOM cluster 5 (Supplementary Table 1) are of interest. The first is* heat shock protein 68 *(*hsp68*), which is a JNK pathway inducible stress response gene. When* Hsp68* is constitutive overexpressed,* D. melanogaster* was protected against oxidative damage and paraquat-induced lethality. In turn, this overexpression led to an approximate 15% increase in median and maximum life span [70]. Another gene in this SOM cluster is the caspase* Death associated molecule related to Mch2 *(*Damm*), which is found at high levels in adults [71]. Changes in caspase activity correlated to age-related apoptosis may play a role in muscle degeneration [72]. In a study of age related changes in the transcriptome in specific body regions,* Damm* was upregulated, suggesting a relationship between proteasome activation and apoptosis in age-related muscle degeneration [10].

There were a number of noteworthy patterns in gene expression in the present study. Two important patterns are described in this paragraph and the third is described in the next paragraph. Firstly, there is a general trend in the results in the present study in which gene clusters that change in mean level of expression as a function of age start to change relatively early in life (e.g., Figure 2). A related observation was made in a metagenomic study comparing life time gene express in* C. elegans* and* D. melanogaster* [3]. Apparently, the gene sets that characterize aging begin to become manifest early in adult life. Secondly, at the last three time points in the present study (65, 72, and 79 days in a cage) there is a tendency for pathways per se to shift in pattern of gene expression. This observation is described in the second paragraph of Results, Section 3.2 *Temporal Gene Set Enrichment Analysis (GSEA).* Apparently our very large cohort sizes enabled us to sample a sufficient number of exceptionally old flies for microarray analysis, and only these ages exhibited shifts in pathway-wide patterns of gene expression.

A third noteworthy pattern of gene expression in the present study is the change in variance in immune system genes as a function of age. The variance in gene expression as a function of age was analyzed in the present study for two sets of genes: immune function genes and a random set of genes. The random set of genes was intended to be representative of much of the genome apart from immune system genes. This comparison of focal interest (immunity versus random set of genes) considered the heterogeneity, specifically variance, of gene expression not mean values per se. No change in mean gene expression was observed for a random set of genes in the present study (Figure 5). This might be expected, as only approximately 10% of the genes in the* Drosophila* genome were found to exhibit a significant change in gene expression in our study. There was an increase in the variation in immune system gene expression within cages (Figure 3) compared to the random sample of genes (Figure 5). This result is consistent with the general observation of genomic instability as a hallmark of aging. Presumably, mutations tend to increase the variation in gene expression as some genes are regulated normally whereas other genes exhibit impaired control of gene expression. Epigenetic alteration as a hallmark of aging [4] is also consistent with our observation of an increase in the variance of gene expression as a function of age. There was no added increase in the variation in gene expression when samples from the different cages were mixed for analysis (among cages, Figure 4) compared to the variation in gene expression when the cages were analyzed separately (Figure 3). This result indicates that the environment between boxes (cages) did not vary substantially. Overall, the analysis of immune function genes in the present study revealed that there was an increase in the variation of gene expression in this class of genes (Figures 4 and 5). The pattern of gene expression change in immune function genes as flies age, an increase in gene expression variation, is compatible with the hypothesis of a loss of normal regulation of gene expression. We focused on immune system genes in our analysis of genes that tend to increase in variance in gene expression as a function of age, but other sets of genes could exhibit a similar age-dependent pattern of change in gene expression. A question arising from the observation of increasing variance in gene expression is what causes immune function genes, or other genes, to be especially liable to loss of control of gene expression as a function of age.

## 5. Conclusion

The present study has provided evidence about the identity of differentially expressed genes that are associated with aging in* D. melanogaster*. Immune function genes were identified as a prominent class of such genes in our study which is consistent with a large body of that data has accumulated to support the inflammaging hypothesis of aging [73]. Our results suggest that loss of control of gene expression of immune function genes is part of the aging process. In conclusion, the individual genes identified by changing expression in the present study encompass a range of targets for future research. Moreover, the clusters of covarying differentially expressed genes identified in the present study could be valuable for future studies of aging as a complex phenotype.

## Supplementary Material

The supplemental tables 1, 2, and 3, are the data used for analysis in this study. Supplemental table 1 presents the data used for the SOM clustering analysis. Supplemental table 2 presents the data used to perform the GSEA for the significantly up-regulated genes, whereas the significantly down-regulated genes are presented in supplemental table 3.

## Figures and Tables

**Table 1:** Differential expression results for cDNA microarray compared to qRT-PCR. Gene expression data from qRT-PCR was normalized using *Rp49*. The fold change is reported as relative to control day 2. Negative numbers represent a downregulation of gene expression at that time point, whereas positive numbers represent an upregulation of gene expression at that time point. *Nplp3* = *Neuropeptide-like precursor 3* and *Mtk* = *Metchnikowin*.

	Day 16		Day 79	
	Microarray	qRT-PCR	Microarray	qRT-PCR
*Retinin *	−3.01	−4.59	−6.45	−18.23
*Nplp3 *	−2.25	−5.07	−4.67	−15.48
*Mtk *	4.80	4.51	12.19	20.32
*CG11671 *	3.58	5.46	7.25	18.86

**Table 2:** PANTHER analysis of significantly differentially expressed genes across times per SOM cluster across all time points. The SOM clusters are shown from clusters 0 to 8 (left to right) across the top of the table. The numbers in the columns indicate the total number of genes found in this group from the gene identification numbers (IDs) able to be mapped in PANTHER. Some genes may be represented in more than one GO category. ^∗^The numbers in the table are the number of unmapped IDs.

Category name (gene ontology accession number)	Cluster								
	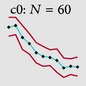	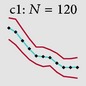	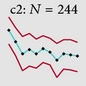	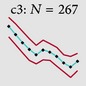	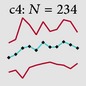	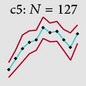	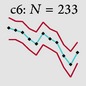	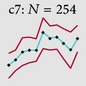	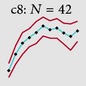
	31^∗^	13^∗^	38^∗^	31^∗^	39^∗^	12^∗^	16^∗^	36^∗^	6^∗^
Cell communication (GO:0007154)	2	15	44	34	37	13	18	37	5
Cellular process (GO:0009987)	3	20	53	43	58	2	25	48	6
Transport (GO:0006810)	4	7	39	2	32	7	29	20	4
Cellular component organization (GO:0016043)	1	7	6	8	19	7	6	7	0
System process (GO:0003008)	2	9	29	35	30	9	15	26	3
Reproduction (GO:0000003)	2	4	8	7	7	3	3	10	0
Response to stimulus (GO:0050896)	2	6	11	8	18	8	9	22	3
Developmental process (GO:0032502)	2	11	16	25	32	8	15	15	2
Metabolic process (GO:0008152)	18	33	94	121	81	37	109	84	10
Immune system process (GO:0002376)	4	6	24	14	26	12	16	30	2
Cell cycle (GO:0007049)	0	5	9	8	15	6	9	13	1
Cell adhesion (GO:0007155)	0	5	11	8	15	3	8	12	2
Apoptosis (GO:0006915)	0	1	9	3	7	6	6	9	0
Generation of precursor metabolites & energy (GO:0006091)	0	1	7	12	3	3	11	3	1
Homeostatic process (GO:0042592)	0	0	1	3	2	1	0	0	0
Localization (GO:0051179)	0	0	0	2	3	0	0	0	0

**Table 3:** Number of genes significantly differentially expressed across time from SOM clustering analysis. The number in the upper left-hand corner of the SOM is the cluster number. The other number in the SOM is the number of genes in that cluster. To be included in the cluster, a gene only needs to be significantly different at any one time point versus the day 2 control. The numbers in the table represent the number of significantly differentially regulated genes at that time point.

Cluster	Days in box										
	9	16	23	30	37	44	51	58	65	72	79
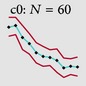	2	6	23	30	57	59	60	59	60	60	60
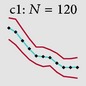		12	35	70	93	101	97	114	111	113	113
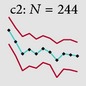		1	45	25	47	24	28	67	42	58	68
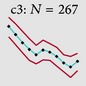		11	39	81	130	95	114	146	228	240	213
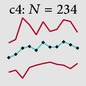		1	26	26	10	51	12	14	48	50	11
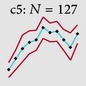		3	42	67	73	118	110	114	86	56	115
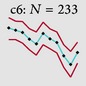		1	2	1	10	1	4	5	74	187	55
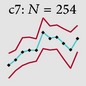		2	20	15	12	143	90	97	57	51	90
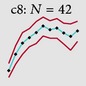		9	37	41	42	42	42	42	42	40	42

Total	2	46	269	356	474	634	557	658	746	855	767

**Table 4:** Distribution of genes differentially regulated at day 79 from PANTHER analysis. The last time point sampled in the analysis was day 79, which represents the oldest living females. The numbers in the table are the number of genes for each SOM that are either upregulated, downregulated, or unaffected at day 79 compared to the day 2 control time point. The numbers in parentheses are the % of genes that are either upregulated, downregulated, or unaffected at day 79 compared to the day 2 control time point. The genes found in SOM clusters 0 and 8 are 100% affected at day 79.

SOM class	Total number of genes	Upregulated	Downregulated	Unaffected
**0**	**60**	**0 (0)**	**60 (100)**	**0 (0)**
1	120	0 (0)	113 (94.2)	7 (5.8)
2	244	1 (0.41)	67 (27.5)	176 (72.1)
3	267	0 (0)	213 (79.8)	54 (20.2)
4	234	11 (4.7)	0 (0)	223 (95.3)
5	127	115 (90.6)	0 (0)	12 (9.4)
6	233	1 (0.43)	54 (23.2)	178 (76.4)
7	254	90 (35.4)	0 (0)	164 (64.6)
**8**	**42**	**42 (100)**	**0 (0)**	**0 (0)**

Total	1581	260 (16.4)	507 (32.1)	814 (51.5)

**Table 5:** Gene ontologies (GO) derived from GSEA of temporal gene expression as compared to day 2 in the box. The first column is the normalized enrichment score (NES), the second column is the name of the GO group, and columns represented by 9–79 are days in the box. The numbers presented in the table are the median fold changes in gene expression. Missing fold change values were replaced by zeroes. The data is ranked and the False Discovery Rate (FDR) cutoff was set at 0.10.

Sum(NES)	Name	9	16	23	30	37	44	51	58	65	72	79
27.7624	ANTIBACTERIAL_HUMORAL_RESPONSE_GO:0019731	0	1.93	2.412	2.374	2.586	2.443	2.544	2.609	2.581	2.701	2.806
26.4471	DEFENSE_RESPONSE_TO_GRAM-POSITIVE_BACTERIUM_GO:0050830	0	2.056	2.219	2.224	2.481	2.491	2.368	2.564	2.528	2.395	2.572
25.3498	DEFENSE_RESPONSE_TO_GRAM-NEGATIVE_BACTERIUM_GO:0050829	0	0	2.368	2.366	2.52	2.446	2.452	2.584	2.586	2.609	2.691
25.1411	DEFENSE_RESPONSE_TO_FUNGUS_GO:0050832	0	2.091	2.289	2.1	2.349	2.441	2.438	2.375	2.173	2.292	2.27
23.1763	DEFENSE_RESPONSE_TO_BACTERIUM_GO:0042742	0	0	2.204	2.278	2.318	2.193	2.258	2.372	2.342	2.437	2.411
22.4411	DEFENSE_RESPONSE_GO:0006952	0	2.032	2.023	2.23	2.033	2.506	2.603	2.392	1.961	0	2.333
16.1288	RESPONSE_TO_STRESS_GO:0006950	0	2.059	2.199	2	0	1.866	1.917	0	2.071	0	2.03
7.8628	CELL_CYCLE_GO:0007049	0	0	0	0	0	0	0	0	2.001	1.974	1.912
7.5424	ANTIFUNGAL_HUMORAL_RESPONSE_GO:0019732	0	0	0	0	0	1.858	0	0	0	1.945	1.847
5.9188	INNATE_IMMUNE_RESPONSE_GO:0045087	0	0	0	0	0	2.05	0	0	0	0	1.919
3.9333	CHROMATIN_SILENCING_GO:0006342	0	0	0	0	0	0	0	0	1.979	1.954	0
3.7802	CYTOKINESIS_GO:0000910	0	0	0	0	0	0	0	0	1.891	1.889	0
2.2342	DNA_REPLICATION_GO:0006260	0	0	0	0	0	0	0	0	0	2.234	0
2.0984	POSITIVE_REGULATION_OF_ANTIFUNGAL_PEPTIDE_BIOSYNTHETIC_PROCESS_GO:0006967	0	0	0	0	0	0	0	0	0	2.098	0
2.0353	EGGSHELL_CHORION_GENE_AMPLIFICATION_GO:0007307	0	0	0	0	0	0	0	0	0	2.035	0
2.0003	ESTABLISHMENT_OR_MAINTENANCE_OF_CELL_POLARITY_GO:0007163	0	0	0	0	0	0	0	0	0	2	0
1.9647	FEMALE_MEIOSIS_CHROMOSOME_SEGREGATION_GO:0016321	0	0	0	0	0	0	0	0	0	1.965	0
1.9552	DNA-DEPENDENT_DNA_REPLICATION_INITIATION_GO:0006270	0	0	0	0	0	0	0	0	0	1.955	0
1.9529	CELLULARIZATION_GO:0007349	0	0	0	0	0	0	0	0	0	1.953	0
1.9523	CHROMOSOME_CONDENSATION_GO:0030261	0	0	0	0	0	0	0	0	0	1.952	0
1.9084	PROGRAMMED_CELL_DEATH_GO:0012501	0	0	0	0	0	0	1.908	0	0	0	0
1.8628	JAK-STAT_CASCADE_GO:0007259	0	0	0	0	0	0	0	0	0	1.863	0
1.861	R8_CELL_FATE_SPECIFICATION_GO:0045464	0	0	0	0	0	0	0	0	1.861	0	0
1.8558	POSITIVE_REGULATION_OF_TOLL_SIGNALING_PATHWAY_GO:0045752	0	0	0	0	0	1.856	0	0	0	0	0
1.852	CELL_FATE_DETERMINATION_GO:0001709	0	0	0	0	0	0	0	0	0	1.852	0
1.8439	CYTOPLASMIC_TRANSPORT,_NURSE_CELL_TO_OOCYTE_GO:0007303	0	0	0	0	0	0	0	0	0	1.844	0
1.8355	OVARIAN_NURSE_CELL_TO_OOCYTE_TRANSPORT_GO:0007300	0	0	0	0	0	0	0	0	0	1.835	0
1.8353	IMMUNE_RESPONSE_GO:0006955	0	0	0	0	0	1.835	0	0	0	0	0
1.8186	PRE-REPLICATIVE_COMPLEX_ASSEMBLY_GO:0006267	0	0	0	0	0	0	0	0	0	1.819	0
1.8163	RESPONSE_TO_PHEROMONE_GO:0019236	0	1.816	0	0	0	0	0	0	0	0	0
1.8026	CILIUM_ASSEMBLY_GO:0042384	0	1.803	0	0	0	0	0	0	0	0	0
1.7815	PEPTIDOGLYCAN_CATABOLIC_PROCESS_GO:0009253	0	0	0	0	0	1.781	0	0	0	0	0
−1.7325	SENSORY_PERCEPTION_OF_CHEMICAL_STIMULUS_GO:0007606	0	0	0	0	0	0	0	0	0	−1.73	0
−1.814	OOGENESIS_GO:0048477	0	0	0	0	0	0	−1.81	0	0	0	0
−1.8205	CELL_WALL_MACROMOLECULE_CATABOLIC_PROCESS_GO:0016998	0	0	0	0	0	0	−1.82	0	0	0	0
−1.8445	MITOCHONDRIAL_ELECTRON_TRANSPORT,_CYTOCHROME_C_TO_OXYGEN_GO:0006123	0	0	0	0	0	0	0	0	0	−1.85	0
−1.8479	PROTON_TRANSPORT_GO:0015992	0	0	0	0	0	0	0	0	0	−1.85	0
−1.8508	SARCOMERE_ORGANIZATION_GO:0045214	0	0	0	0	0	0	0	−1.85	0	0	0
−1.8693	REGULATION_OF_RHO_PROTEIN_SIGNAL_TRANSDUCTION_GO:0035023	0	0	0	0	0	−1.87	0	0	0	0	0
−1.9418	REGULATION_OF_SYNAPSE_STRUCTURE_AND_ACTIVITY_GO:0050803	0	−1.94	0	0	0	0	0	0	0	0	0
−1.9713	MITOTIC_SPINDLE_ELONGATION_GO:0000022	−1.97	0	0	0	0	0	0	0	0	0	0
−2.0548	COURTSHIP_BEHAVIOR_GO:0007619	0	0	−2.06	0	0	0	0	0	0	0	0
−2.0802	MESODERM_DEVELOPMENT_GO:0007498	0	−2.08	0	0	0	0	0	0	0	0	0
−2.2744	MITOCHONDRIAL_ELECTRON_TRANSPORT,_NADH_TO_UBIQUINONE_GO:0006120	0	0	0	0	0	0	0	0	0	−2.27	0
−3.5887	TRANSPORT_GO:0006810	0	0	0	0	0	0	0	0	−1.78	−1.8	0
−3.7605	PROTEOLYSIS_GO:0006508	0	0	0	0	0	0	0	0	−1.82	−1.94	0
−3.7839	FLIGHT_BEHAVIOR_GO:0007629	0	−1.94	0	0	0	0	−1.84	0	0	0	0
−3.8231	NEUROPEPTIDE_SIGNALING_PATHWAY_GO:0007218	0	0	−1.85	−1.98	0	0	0	0	0	0	0
−3.981	SYNAPTIC_VESICLE_EXOCYTOSIS_GO:0016079	0	−2.09	−1.9	0	0	0	0	0	0	0	0
−4.7191	TRANSLATION_GO:0006412	−2.52	0	0	0	0	0	0	0	0	−2.2	0
−5.5074	VITELLOGENESIS_GO:0007296	0	0	0	0	0	−1.86	−1.86	0	0	−1.79	0
−5.6526	MYOFIBRIL_ASSEMBLY_GO:0030239	0	−1.94	−1.86	−1.86	0	0	0	0	0	0	0
−5.9978	CALCIUM_ION_TRANSPORT_GO:0006816	0	−2.04	0	0	0	−1.94	−2.02	0	0	0	0
−7.4059	SEX_DIFFERENTIATION_GO:0007548	0	0	0	0	0	0	0	0	−1.83	−1.75	−1.9
−7.5705	LIPID_METABOLIC_PROCESS_GO:0006629	0	0	0	0	0	0	0	0	−1.89	−1.83	−1.91
−11.3157	DEACTIVATION_OF_RHODOPSIN_MEDIATED_SIGNALING_GO:0016059	0	−1.98	−2	0	0	0	−1.84	0	−1.79	0	−1.85
−13.8505	CHORION-CONTAINING_EGGSHELL_FORMATION_GO:0007304	0	0	0	0	0	−2.08	−1.97	−1.92	−2.05	−1.79	−2.04
−13.8792	MULTICELLULAR_ORGANISMAL_DEVELOPMENT_GO:0007275	0	0	0	0	0	−1.96	−1.96	−1.85	−2.1	−1.88	−2.1
−17.7278	VITELLINE_MEMBRANE_FORMATION_INVOLVED_IN_CHORION-CONTAINING_EGGSHELL_FORMATION_GO:0007305	0	0	0	−1.89	−1.89	−1.95	−1.97	−1.93	−1.99	−2.04	−2.07
−19.9978	EGGSHELL_CHORION_ASSEMBLY_GO:0007306	0	0	0	−2.06	−2.15	−2.32	−2.23	−2.17	−2.32	−2.17	−2.29
−20.3805	PHOTOTRANSDUCTION_GO:0007602	0	−2.4	−2.04	−2.12	−2.18	0	−1.89	−1.99	−2.01	−1.9	−1.92

**Table 6:** Kyoto Encyclopedia of Genes and Genomes (KEGG) pathways identified using GSEA of temporal gene expression as compared to day 2 in the box. The first column is the normalized enrichment score (NES), the second column is the name of the KEGG pathway, and columns represented by 9–79 are days in the box. The numbers presented in the table are the median fold changes in gene expression. Missing fold change values were replaced by zeroes. The data is ranked and the False Discovery Rate (FDR) cutoff was set at 0.10.

Sum(NES)	Name	9	16	23	30	37	44	51	58	65	72	79
3.9903	LIMONENE_AND_PINENE_DEGRADATION_00903	1.905	0	0	0	0	2.09	0	0	0	0	0
2.1169	DNA_REPLICATION_03030	0	0	0	0	0	0	0	0	0	2.117	0
1.9396	MISMATCH_REPAIR_03430	0	0	0	0	0	0	0	0	0	1.94	0
1.8761	PORPHYRIN_AND_CHLOROPHYLL_METABOLISM_00860	1.876	0	0	0	0	0	0	0	0	0	0
1.8689	PENTOSE_AND_GLUCURONATE_INTERCONVERSIONS_00040	1.869	0	0	0	0	0	0	0	0	0	0
1.8595	BASE_EXCISION_REPAIR_03410	0	0	0	0	0	0	0	0	0	1.86	0
1.7939	GLYCOSAMINOGLYCAN_BIOSYNTHESIS_-_HEPARAN_00534	0	0	0	0	0	0	0	0	0	1.794	0
0.1672	ASCORBATE_AND_ALDARATE_METABOLISM_00053	1.971	0	0	0	−1.8	0	0	0	0	0	0
0.0946	RETINOL_METABOLISM_00830	1.871	0	0	0	−1.78	0	0	0	0	0	0
−1.7027	FATTY_ACID_BIOSYNTHESIS_00061	0	0	0	0	−1.7	0	0	0	0	0	0
−1.7171	ECM-RECEPTOR_INTERACTION_04512	0	0	0	0	0	0	0	0	0	−1.72	0
−1.7337	GLYCOLYSIS_/_GLUCONEOGENESIS_00010	0	0	0	0	0	0	0	0	−1.73	0	0
−1.741	OTHER_GLYCAN_DEGRADATION_00511	0	0	0	−1.74	0	0	0	0	0	0	0
−1.7658	FOLATE_BIOSYNTHESIS_00790	0	0	0	0	0	0	0	0	0	−1.77	0
−1.7669	ALANINE_ASPARTATE_AND_GLUTAMATE_METABOL_00250	0	0	0	0	0	0	0	0	−1.77	0	0
−1.8355	NEUROACTIVE_LIGAND-RECEPTOR_INTERACTION_04080	0	0	0	0	0	0	0	0	0	0	−1.84
−3.6284	PYRUVATE_METABOLISM_00620	0	0	0	0	0	0	0	0	−1.79	−1.84	0
−3.6518	ARGININE_AND_PROLINE_METABOLISM_00330	0	0	0	0	0	0	0	0	0	−1.87	−1.78
−4.318	OXIDATIVE_PHOSPHORYLATION_00190	−1.91	0	0	0	0	0	0	0	0	−2.41	0
−6.4788	RIBOSOME_03010	−2.54	0	0	0	0	0	0	0	−1.82	−2.12	0
−9.3465	STARCH_AND_SUCROSE_METABOLISM_00500	0	0	0	−1.75	−2.04	0	0	0	−1.85	−1.82	−1.89
−9.5827	GALACTOSE_METABOLISM_00052	0	0	0	−1.92	−1.86	0	0	0	−2.08	−1.97	−1.74
−18.1624	PHOTOTRANSDUCTION_-_FLY_04745	0	−2.28	−2.21	−2.09	−2.07	0	−1.95	−2.02	−1.87	−1.9	−1.77
